# Machine learning based estimation of dynamic balance and gait adaptability in persons with neurological diseases using inertial sensors

**DOI:** 10.1038/s41598-023-35744-x

**Published:** 2023-05-27

**Authors:** Piergiuseppe Liuzzi, Ilaria Carpinella, Denise Anastasi, Elisa Gervasoni, Tiziana Lencioni, Rita Bertoni, Maria Chiara Carrozza, Davide Cattaneo, Maurizio Ferrarin, Andrea Mannini

**Affiliations:** 1 AIRLab, IRCCS Fondazione Don Carlo Gnocchi ONLUS, 50143 Florence, Italy; 2 Scuola Superiore Sant’Anna, Istituto di BioRobotica, 56025 Pontedera, Italy; 3 LAMoBIR and LaRiCE, IRCCS Fondazione Don Carlo Gnocchi ONLUS, 20148 Milan, Italy; 4 Dipartimento di Fisiopatologia Medico-Chirurgica e dei Trapianti, Università di Milano, 20122 Milan, Italy

**Keywords:** Predictive markers, Rehabilitation, Multiple sclerosis, Parkinson's disease, Stroke, Biomedical engineering

## Abstract

Poor dynamic balance and impaired gait adaptation to different contexts are hallmarks of people with neurological disorders (PwND), leading to difficulties in daily life and increased fall risk. Frequent assessment of dynamic balance and gait adaptability is therefore essential for monitoring the evolution of these impairments and/or the long-term effects of rehabilitation. The modified dynamic gait index (mDGI) is a validated clinical test specifically devoted to evaluating gait facets in clinical settings under a physiotherapist’s supervision. The need of a clinical environment, consequently, limits the number of assessments. Wearable sensors are increasingly used to measure balance and locomotion in real-world contexts and may permit an increase in monitoring frequency. This study aims to provide a preliminary test of this opportunity by using nested cross-validated machine learning regressors to predict the mDGI scores of 95 PwND via inertial signals collected from short steady-state walking bouts derived from the 6-minute walk test. Four different models were compared, one for each pathology (multiple sclerosis, Parkinson’s disease, and stroke) and one for the pooled multipathological cohort. Model explanations were computed on the best-performing solution; the model trained on the multipathological cohort yielded a median (interquartile range) absolute test error of 3.58 (5.38) points. In total, 76% of the predictions were within the mDGI’s minimal detectable change of 5 points. These results confirm that steady-state walking measurements provide information about dynamic balance and gait adaptability and can help clinicians identify important features to improve upon during rehabilitation. Future developments will include training of the method using short steady-state walking bouts in real-world settings, analysing the feasibility of this solution to intensify performance monitoring, providing prompt detection of worsening/improvements, and complementing clinical assessments.

## Introduction

Healthy people easily adapt to environmental perturbations: they recover from external impulses and learn walking dynamics in different contexts. Locomotion requires a continuous modulation of the coordination within and between limbs to maintain the equilibrium during progression and to accommodate demands coming from the real-world environment (e.g., passing from straight-line walking to walking over an obstacle)^1^. To achieve this goal, the central nervous system exploits the sensorimotor control that conveys and integrates visual, proprioceptive, and vestibular sensory inputs to detect deviations from the upright posture and generate the appropriate muscle response to correct these deviations^1, 2^. Because sensorimotor feedback is altered in persons with neurological disorders (PwND), dynamic balance (i.e. the ability to maintain balance while moving the body) and its adaptability to environmental changes are impaired^3^, leading to a high risk of falling. In fact, the most commonly found predictors of falls are gait impairments and balance disorders^4^. Thus, many dynamic balance assessment tools have been developed, including clinical scales^5^, quantitative gait markers^6^, posturography^7^, fall prevention protocols^8^ and single- and dual-task tests^9^. Regarding clinical scales, the Berg balance scale^10^, the MiniBESTest^10^, the timed up and go (TUG)^11^, and the modified dynamic gait index (mDGI)^12, 13^ are among the most commonly used in clinical practice to measure balance in PwND. Compared to the Berg scale, which does not assess dynamic balance during locomotion, and the TUG and MiniBESTest, which evaluate this aspect in a few functional tasks (i.e., one task in TUG and five tasks in MiniBESTest), the mDGI is specifically devoted to measuring an individual’s capacity to maintain their balance and adapt their gait in the presence of various environmental demands, essential to perform daily-life locomotor activities without falling. The mDGI assesses eight facets of gait; it evaluates distance, temporal, ambient, terrain, physical load, attention, and postural transitions, which are representative of the environmental demands of a walking human. The highest possible score on the mDGI is 64 points. The mDGI has been extensively validated in subjects with mobility impairments^14, 15^ and in different pathological cohorts such as stroke (ST), vestibular dysfunction, multiple sclerosis (MS), traumatic brain injuries, and Parkinson’s disease (PD)^12, 13^. Recently, Torchio et al. provided cut-off values to identify PwND with no or minimal risk of falls (mDGI score $> 49$) and PwND with high risk of falls (mDGI score $\le 29$)^16^. The minimal detectable change in mDGI is 5 points in PwND^14, 15^.

A previous study of the eight items of the mDGI found a strong correlation between the mDGI score and instrumentally-derived indexes^17^, suggesting that kinematic determinants can predict dynamic balance. Despite these encouraging results, the assessment of equilibrium and gait adaptation to different environments is still performed only by specialised personnel in clinical settings and requires specific tools, such as obstacles or stairs. Such requirements, in turn, limits the number of possible assessment sessions, while a frequent monitoring would better track the possible change caused by pathology course or rehabilitation/pharmacological interventions. In this respect, the opportunity of predicting dynamic balance and locomotor adaptability by using wearable sensors during repeated short (i.e. 10 strides) steady-state walking bouts, easily performed during daily living also by PwND (e.g. during a stroll alone or with a caregiver), would represent a first step in increasing the monitoring frequency and complement the periodic in-clinic evaluations^18, 19^.

As a preliminary test of this opportunity, this study implemented an interpretable machine learning (ML) model targeting the mDGI score by using inertial measurements units (IMUs) to collect data during short steady-state walking bouts of a 6-minute walk test (6MWT). After statistically confirming the association between instrumental variables and the mDGI score, we hypothesised that ML models based on such variables can predict dynamic balance and gait adaptability (i.e., mDGI scores) in PwND, including multiple sclerosis (MS), Parkinson’s disease (PD), or stroke (ST). If this hypothesis is confirmed, the results of the present study could offer a clear starting for assessing the feasibility of this approach in daily life. Determinants of gait temporal aspects, intensity, smoothness, stability, symmetry, and regularity were extracted from a 3-IMU set-up. Then, a regularized Elastic-Net (EN) regression was developed using a nested cross-validation approach. This pipeline was repeated for the multi-pathological cohort (MP) and the single pathology cohorts (SP$_{MS}$, SP$_{PD}$, and SP$_{ST}$). Furthermore, we integrated the best performing solution model with a Shapley-values based explainability technique (SHapley Additive exPlanations, SHAP^20, 21^).

## Results

### Cohort

The pooled cohort—95 PwND, F = 43, median age = 60 years [IQR = 19]—resulted in a median 6MWT score of 346 m [IQR = 21] and a median mDGI of 46 points [IQR = 21] (Table 1). For the MS, PD and ST groups, the median disease duration corresponded to 19, 4, and 7 years, respectively. The median 6MWT was 316 [IQR = 182], 332 [IQR = 194] and 372 [IQR = 152] m, and the median mDGI score was 40 [IQR = 21], 46 [IQR = 29] and 50 [IQR = 14], respectively. Following the cut-off values defined by Torchio et al.^16^, 39 of the 95 participants (41%) had a low/minimal fall risk (mDGI score > 49), while 17 (18%) had a high fall risk (mDGI score $\le$ 29). The number (percentage) of individuals with small/minimal fall risk was 18 (35%) for MS, 17 (59%) for PD, and 4 (27%) for the ST group. The number (percentage) of individuals at high fall risk was 7 (14%) for MS, 3 (10%) for PD, and 4 (27%) for the ST group.

**Table 1:** MS: Multiple sclerosis; PD: Parkinson’s disease; ST: stroke; 6MWT: 6-minute walk test; mDGI: modified dynamic gait index; MP: multi-pathology; SP: single pathology for numerical independent variables, median and interquartile range was reported using brackets. On the other hand, categorical independent variables were reported by means of the number of occurrences of the positive class (e.g. ‘having a bilateral support’) and the related percentage in parenthesis.

	MP (N= 95)	SP: *MS* (N = 51)	SP: *PD* (N = 25)	SP: *ST* (N = 19)
Age, years	62 [19]	54 [22]	68 [15]	71 [14]
Sex (F)	47 (49.4)	23 (45.1)	13 (52.0)	11 (57.9)
Disease duration, years	12 [15]	19 [18]	4 [9]	7 [7]
Assistive device				
Monolateral	15 (15.7)	10 (19.6)	4 (16.0)	1 (5.2)
Bilateral	19 (20.0)	14 (27.5)	3 (12.0)	2 (10.5)
6MWT score, m	346 [169]	316 [182]	332 [194]	372 [152]
mDGI	46 [21]	40 [21]	46 [29]	50 [14]

### Univariate analysis

Preliminary univariate Spearman’s correlation showed that the 6MWT was significantly associated with the mDGI ($p < 0.001$) for the pooled and the individual cohorts (Table 2). In the pooled cohort, longer durations of stride and double support times resulted in a decrease in the mDGI. A positive association with dynamic balance was found for swing and single support time. The same trends were found in the MS and PD groups. Conversely, the ST group exhibited this behavior only for $T_{d,support}$ and $T_{s,support}$. Gait regularity (in the form of step and stride regularity) was positively associated with the mDGI score ($p < 0.01$) for all axes in the pooled and MS cohorts, and on the vertical axes for the PD group. Moreover, mediolateral (ML) stride regularity was positively correlated with the mDGI in PD patients. Smoothness of walking, expressed as an improved harmonic ratio (iHR), resulted in a positive relationship with the mDGI for all three axes in the MS, PD, and pooled cohorts and in the vertical (VT) direction for patients with ST. Root mean squared acceleration values were significantly associated with the mDGI for all groups and all axes. Lyapunov exponents in the AP direction of the pooled and MS cohorts exhibited a negative association with the outcome ($p < 0.01$). The single-pathology group, specifically the MS and PD groups, demonstrated an inversely significant relationship between normalised vertical jerk and the mDGI values. Furthermore, in people with ST and MS, the mDGI was inversely associated with age and disease duration. The presence of either bilateral or monolateral support significantly reduced the mDGI performance, particularly in the MS and PD groups.

**Table 2:** As test statistics, Spearman’s correlations were reported for numerical independent variables, Mann–Whitney’s U for two-class categorical variables (e.g. gender) and Kruskal–Wallis $\chi (DoF)$ for multi-class categorical variables (e.g. presence of assistive device).

	MP (N = 95)		SP: *MS* (N = 51)		SP: *PD* (N = 25)		SP: *ST* (N = 19)	
	*p* value	Test statistics	*p* value	Test statistics	*p* value	Test statistics	*p* value	Test statistics
Age	0.653	− 0.047	**< 0.05**	**− 0.290**	0.715	0.077	**< 0.05**	**− 0.488**
Sex	**< 0.05**	**851.000**	0.421	279.500	0.205	54.500	0.492	35.000
Disease duration	0.073	− 0.185	**< 0.05**	**− 0.334**	**< 0.05**	**0.403**	**< 0.01**	**− 0.616**
Assistive device	**< 0.001**	**46.990(2)**	**< 0.001**	**29.409(2)**	**< 0.01**	**11.532(2)**	0.084	4.960(2)
6MWT score	**< 0.001**	**0.869**	**< 0.001**	**0.851**	**< 0.001**	**0.839**	**< 0.001**	**0.830**
T$_{stride}$	**< 0.001**	**− 0.464**	**< 0.001**	**− 0.544**	**< 0.05**	**− 0.469**	0.700	0.095
T$_{swing}$	**< 0.001**	**0.494**	**< 0.001**	**0.523**	**< 0.01**	**0.626**	0.592	0.131
T$_{d,support}$	**< 0.001**	**− 0.593**	**< 0.001**	**− 0.583**	**< 0.001**	**− 0.738**	**< 0.05**	**− 0.516**
T$_{s,support}$	**< 0.001**	**0.589**	**< 0.001**	**0.579**	**< 0.001**	**0.712**	**< 0.05**	**0.548**
T$_{step}$	0.080	− 0.181	0.170	− 0.195	**< 0.01**	**− 0.561**	0.884	− 0.036
Step regularity$_{ap}$	**< 0.001**	**0.483**	**< 0.001**	**0.515**	0.135	0.307	**< 0.05**	**0.507**
Stride regularity$_{ap}$	**< 0.01**	**0.326**	0.052	0.274	0.066	0.373	0.412	0.200
Step regularity$_{ml}$	**< 0.01**	**0.270**	**< 0.05**	**0.300**	0.099	0.338	0.809	0.060
Stride regularity$_{ml}$	**< 0.001**	**0.550**	**< 0.001**	**0.590**	**< 0.01**	**0.520**	0.404	0.203
Step regularity$_{vt}$	**< 0.001**	**0.548**	**< 0.01**	**0.395**	**< 0.001**	**0.734**	0.092	0.397
Stride regularity$_{vt}$	**< 0.001**	**0.504**	**< 0.001**	**0.506**	**< 0.01**	**0.623**	0.771	0.071
CV$_{T,stride}$	**< 0.001**	**− 0.680**	**< 0.001**	**− 0.566**	**< 0.001**	**− 0.711**	**< 0.01**	**− 0.619**
CV$_{T,step}$	**< 0.001**	**− 0.575**	**< 0.01**	**− 0.392**	**< 0.001**	**− 0.655**	**< 0.05**	**− 0.464**
iHR$_{ap}$	**< 0.001**	**0.619**	**< 0.001**	**0.536**	**< 0.001**	**0.769**	0.156	0.339
iHR$_{ml}$	**< 0.001**	**0.569**	**< 0.001**	**0.544**	**< 0.01**	**0.623**	0.235	0.286
iHR$_{vt}$	**< 0.001**	**0.735**	**< 0.001**	**0.624**	**< 0.001**	**0.796**	**< 0.01**	**0.659**
Lyapunov step$_{ap}$	**< 0.01**	**− 0.312**	**< 0.01**	**− 0.386**	0.264	− 0.232	0.232	− 0.288
Lyapunov step$_{ml}$	0.826	− 0.023	0.978	− 0.004	0.835	0.044	0.297	− 0.252
Lyapunov step$_{vt}$	0.157	− 0.146	0.111	− 0.226	0.791	0.056	0.653	− 0.110
RMS Acc.$_{ap}$	**< 0.001**	**0.593**	**< 0.001**	**0.594**	**< 0.01**	**0.531**	**< 0.01**	**0.597**
RMS Acc.$_{ml}$	**< 0.001**	**0.439**	**< 0.001**	**0.472**	0.050	0.397	**< 0.05**	**0.561**
RMS Acc.$_{vt}$	**< 0.001**	**0.673**	**< 0.001**	**0.717**	**< 0.001**	**0.686**	**< 0.05**	**0.508**
Log. Norm. Jerk$_{ap}$	0.782	− 0.029	0.476	− 0.102	0.324	0.206	0.492	− 0.168
Log. Norm. Jerk$_{ml}$	**< 0.01**	**− 0.342**	0.153	− 0.203	0.574	− 0.118	0.800	− 0.062
Log. Norm. Jerk$_{vt}$	**< 0.001**	**− 0.478**	**< 0.001**	**− 0.541**	**< 0.01**	**− 0.610**	0.715	0.090

Dependent variable was set to the mDGI total score.

Significant values are in [bold].

*MP* multi-pathological, *SP* single-pathology, *MS* multiple sclerosis, *PD* Parkinson’s disease, *ST* stroke, *6MWT* 6-minutes walk test, *T* time, *CV* coefficient of variation, *iHR* improved harmonic ratio, *RMS* root mean squared, *AP* antero-posterior, *ML* medio-lateral, *VT* vertical.

### Prediction

**Figure 1:**
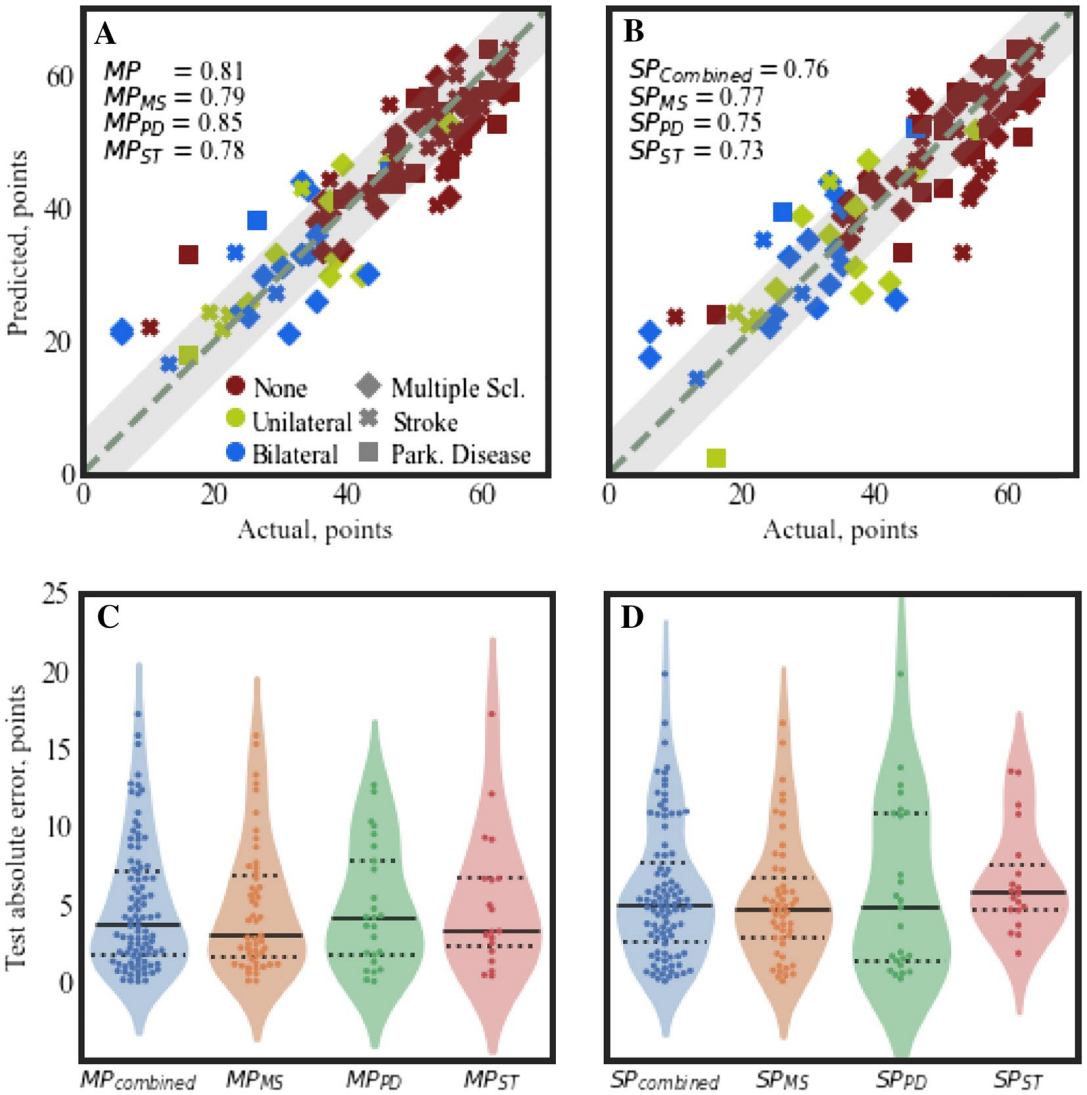
Test predictions plot for the MP model (panel **A**) and the ST models (panel **B**). In both panels, the marker color indicates the type of assistive support while the marker type indicates the pathology. The dashed grey line $y = x$ indicates ideal predictions, while the shaded grey rectangle around it represents a boundary of $\pm 5$ points (MDC). Below, with violin-plots (and superimposed swarm-plots) the test absolute error distributions are represented. The MP model (*MP*) test errors were grouped according to the pathology ($MP_{MS}, \; MP_{PD}, \; MP_{ST}$) and provided in panel (**C**), whilst the SP models ($SP_{combined}$) combined test errors and the respective individual models are reported in panel (**D**).

In the MP model, the optimized EN model resulted in a regressor with a median absolute validation error of 4.07 points [IQR = 0.07] across the 95 leave-one-subject-out (LOSO) outer folds. The aggregated test absolute error was equal to 3.58 points [IQR = 5.41] with a $R^{2} = 0.81$ (Fig. 1, panel A). Thus, 76% of the predictions were within the mDGI’s minimal detectable change of 5 points. By subdividing MP predictions into the three sub-cohorts (Fig. 1, panel C) and computing correlation coefficients within the groups, an $R^{2}$ of 0.79, 0.85, and 0.78 was obtained respectively for the MS, PD, and ST sub-cohorts, respectively. Similarly, individual group test errors resulted in 2.92 [IQR = 5.21], 4.11 [IQR = 6.04], and 3.22 [IQR = 4.41] points for the the MS, PD and ST sub-cohorts, respectively. Single-pathology (SP) models resulted in an overall (aggregated) test error of 4.91 points [IQR = 5.09] and an $R^{2}$ = 0.76 (Fig. 1, panel B). Namely, individual SP models resulted in a median absolute test error of 4.64 points [IQR = 3.86] for the MS group, 4.76 points [IQR = 9.47] for the PD group, and 5.64 points [IQR = 2.92] for the ST group (Fig. 1, panel D). No significant differences (Wilcoxon signed-rank tests) were found between the combined SP models and the MP model or between the individual SP models and their respective groupings of the MP predictions. Models trained using data not derived from inertial sensors resulted in reduced accuracy in both the MP and SP cases. In particular, for the MP and the combined SP models, the $R^{2}$ decreased from 0.81 to 0.56 and from $R^{2}$ = 0.76 to 0.46, respectively (Supplementary Fig. 1). Applying a threshold of 5 to the MP model derived from clinical data only, a classification accuracy of 49% was obtained. Similarly, after selecting individual pathologies from the MP model, the $R^{2}$ decreased from 0.79, 0.85, and 0.78–0.55, 0.75 and 0.22, respectively for the MS, PD, and ST models (Supplementary Fig. 1). In order to assess the model’s predictive performance without the 6MWT score (estimate of walking speed), the pipeline was repeated, removing this information from the training data (Supplementary Fig. 2). This approach resulted in a median absolute error of 5.01 [IQR = 5.6] for the MP model ($R^{2}$ = 0.78), with no significant differences from the MP model with the 6MWT score included (Mann–Whitney, $p > 0.05$).

**Figure 2:**
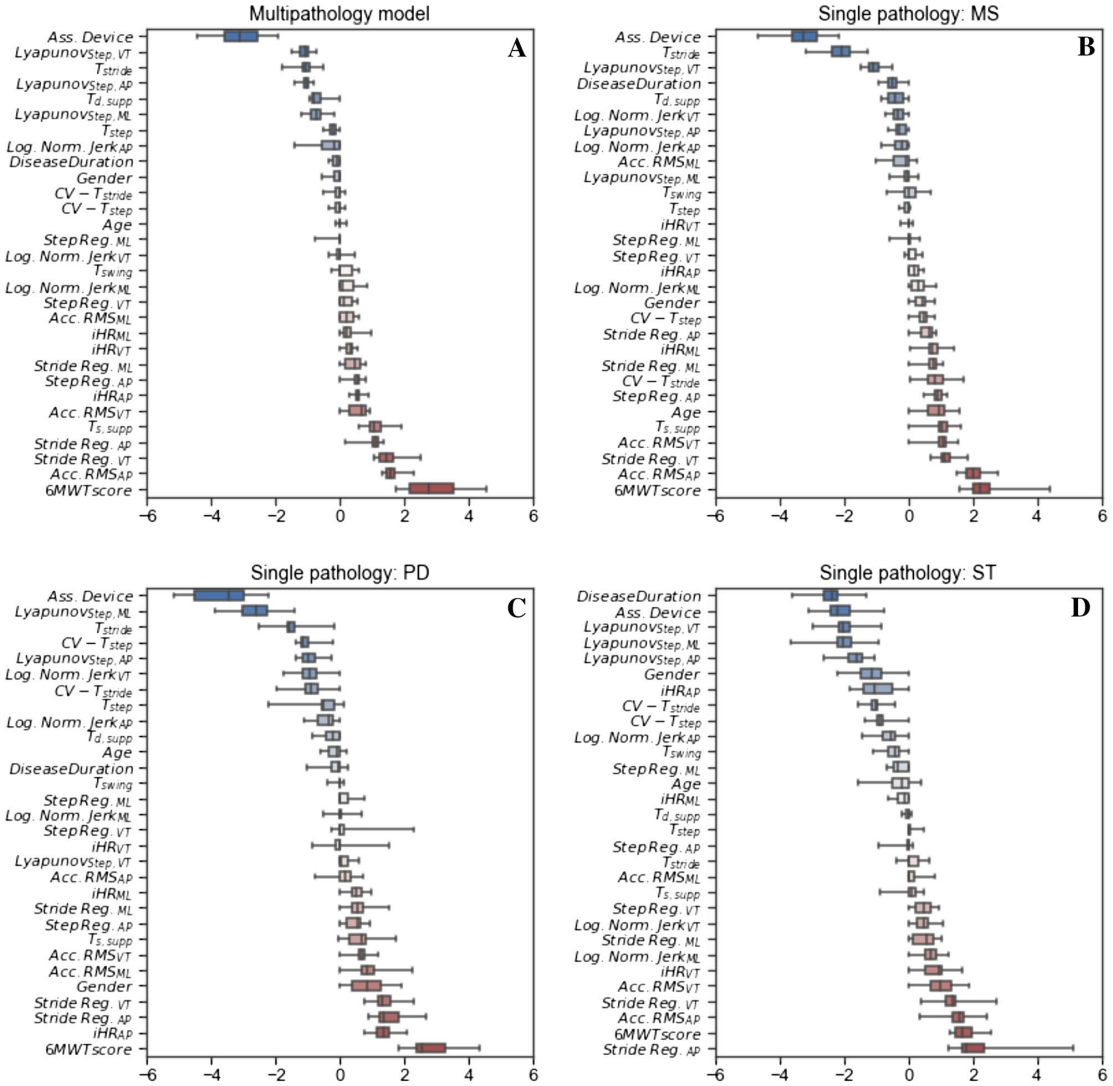
Regression $\beta$ coefficients of the Elastic-Net models for the MP (panel **A**), $SP_{MS}$ (panel **B**), $SP_{PD}$ (panel **C**) and $SP_{ST}$ (panel **D**). Regression coefficients obtained from the models of the outer leave-one-subject-out split are aggregated. Therefore each variable weight is indicated via a box-plot instead than a bar-plot to account for this variability. Features are sorted in ascending order of median $\beta$ values across the folds.

In the MP model (Fig. 2, panel A), the presence of a bilateral or mono-lateral assistive device was the factor that showed the strongest negative association with the outcome. Lyapunov coefficients, calculated on all three axes, were negatively correlated with the mDGI prediction. Additionally, longer $T_{stride}$ and $T_{d,supp}$ was related to a reduced dynamic balance (i.e., lower mDGI score). Walking endurance (i.e., the distance covered in 6 min, as measured with the 6MWT), movement intensity (i.e., *Acc*.*RMS*), stride regularity computed on the AP/VT axes, and duration of the single support phase $T_{s,supp}$ were positively associated with the mDGI scores and, consequently, with a strong ability to maintain dynamic balance. In the $SP_{MS}$ model (Fig. 2, panel B), the presence of an assistive device, longer $T_{stride}$ and longer $T_{d,supp}$ were associated with lower mDGI values. Furthermore, higher values of normalized jerk values and higher Lyapunov exponents in the VT/AP directions resulted in reduced dynamic balance. Increased stride regularity on the three axes, longer $T_{s,supp}$ and higher acceleration values on the AP axes were all related to higher mDGI in the MS group.

In the PD group, the presence of an assistive device was the variable that showed the most negative association with the outcome (Fig. 2, panel C). Lower mDGI scores in people with PD were also negatively impacted by higher values of $CV_{T,step}$, $CV_{T,stride}$ (i.e., step and stride variability) and $T_{stride}$. Conversely, the Lyapunov of the ML/AP axes maintained a strong negative effect on the mDGI in the PD model. People with PD were almost unaffected by changes in Lyapunov coefficients in the vertical direction. Faster acceleration RMS values and higher stride regularity in all three directions were associated with higher mDGI values in the PD model and higher gait symmetry quantified by $iHR_{AP}$ and $iHR_{ML}$.

Notably, in the ST model, the disease duration and the presence of assistive devices were the variables most negatively associated variables with the mDGI. Lyapunov coefficients in all directions affected the balance of participants post-stroke (Fig. 2, panel D). As in the case of participants with PD, the ST cohort exhibited a strong negative association between $CV_{T,step}$ and $CV_{T,stride}$ and the mDGI score. Stride regularity and RMS acceleration values on the AP axes were the features most strongly correlated with the mDGIs of participants post-stroke.

### Interpreting MP model

**Figure 3:**
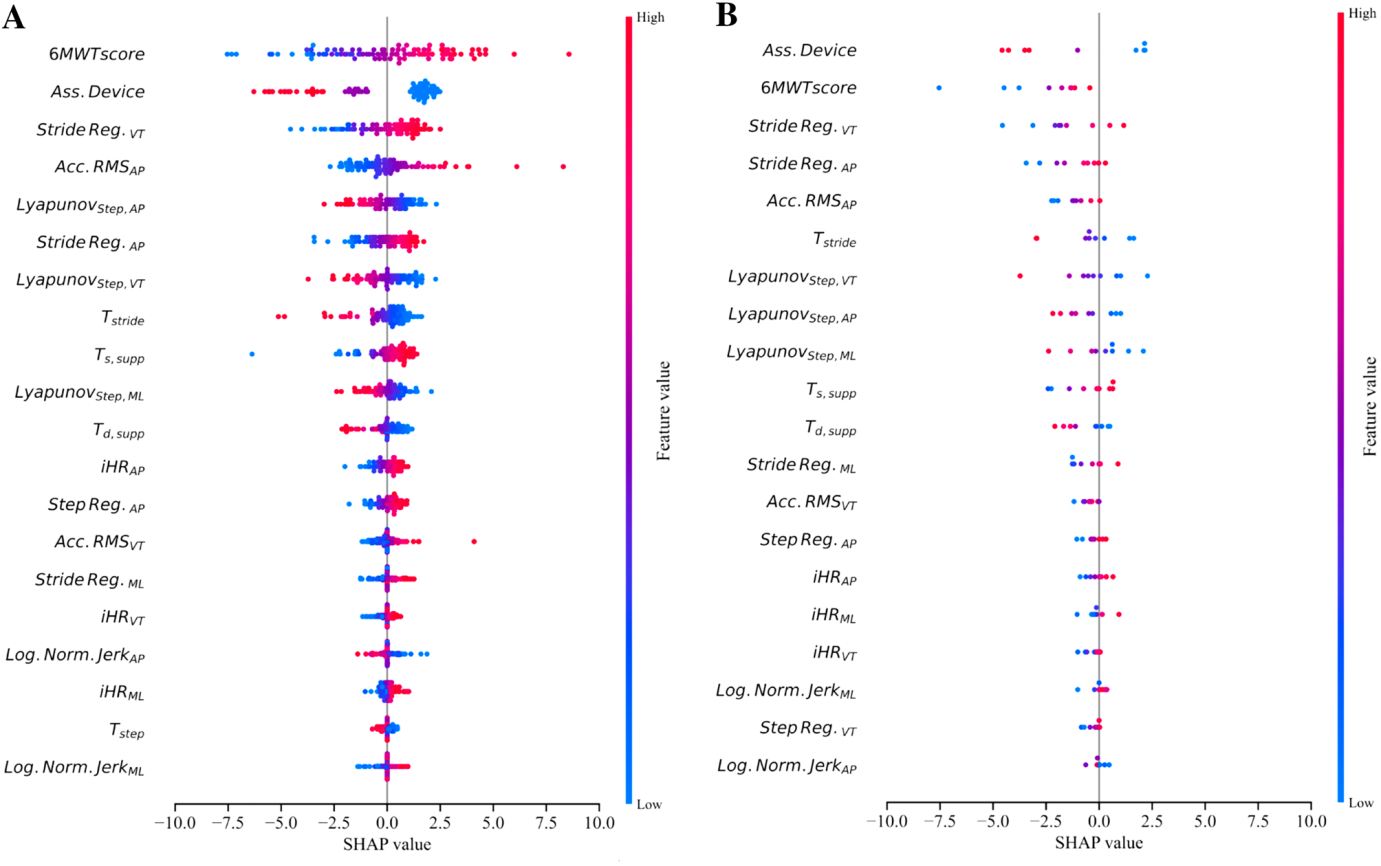
SHAP values of the MP model were computed for all features coalitions repeatedly for each of the outer test folds and aggregated together. In Panel (**A** and **B**) they are presented ordered according to the $mean|Shapley_{i}|$ across all test patients. Respectively, in panel (**A**) all instances are reported whilst on panel (**B**) only instances mis-classified by more than 10 mDGI points are shown. On the x-axes, the individual features normalize contribution on the mDGI values is reported, with the model prediction for each patient being the sum across all features contribution.

The SHAP values of the MP model (Fig. 3) indicated that the strongest mDGI predictor is the 6MWT score. With respect to assistive devices, three distinctive effects are observed: no assistive device (blue), monolateral assistance (purple), and bilateral assistance (pink). Compared to monolateral assistance, the use of bilateral assistive devices resulted in stronger limitations of dynamical capabilities. In conjunction with related regression coefficients, high values of stride regularity on the VT and AP axes positively impacted the mDGI. Moreover, higher values of anteroposterior RMS acceleration contributed to increasing predicted values. The SHAP values confirmed that Lyapunov coefficients on all three axes provided a strong contribution to the mDGI prediction with a negative effect (i.e., higher Lyapunov coefficients are predictive of lower predicted mDGI). Furthermore, faster strides and longer single support periods positively influenced the model predictions. The feature contributions to the prediction of patients with an absolute prediction error larger than 10 points were compared to the contributions of the whole cohort. No systematic differences in feature importance were found between the misclassified and the correctly predicted patients (Fig. 3, panel A and B).

## Discussion

In this study, we trained, optimised, and cross-validated an elastic net regression model capable of predicting the mDGI score from a 3-IMU setup used during short steady-state walking bouts (10 strides each) extracted from the 6MWT. The median [IQR] error was 3.58 [5.38] points, which is lower than the mDGI MDC (i.e., 5 points). Elastic net was chosen as the adopted model because it is a regularised multivariate linear regression and one of the simplest ML models. In particular, regularisation was necessary to address the high dimensionality of the predictors compared to the moderate sample size and to prevent overfitting. Regularisation was chosen instead of statistical feature screening methods to avoid train-test contamination. Lastly, linear regressions such as elastic net also allow for the evaluation of regression coefficients and hence a model-based feature importance assessment. Comparing the developed models with algorithms trained only on features not derived from IMUs (6MWT score and presence of assistive devices) showed that IMU-based features are crucial in the assessment of dynamic balance and gait adaptability. In particular, comparing the classification performances obtained after applying an MDC-based threshold showed how using IMU-related features improved accuracy by 27% (from 49 to 76%), resulting improved classification for 26 out of 95 patients. These encouraging results represent a strong starting point for future studies focusing on the feasibility of this approach in real-world contexts, with the final goal of increasing monitoring frequency and complementing in-clinic assessment.

The multipathology model (MP), trained with a cohort of people with MS, PD, and ST, resulted in the best-performing solution. Unexpectedly, single-pathology (SP) models did not outperform the MP models. The reason may be twofold. First, the PD and ST cohorts were considerably smaller than the MS cohort. Therefore, in people with ST and PD, predictions of models trained on the MP cohort may have been influenced by the presence, in the training set, of the MS patients, who comprised almost 50% of the participants. Second, different neurological impairments could lead to different gait pattern alterations, resulting in comparable effects in terms of dynamic balance and gait adaptability, which may still be interpretable by a MP model. The latter was further confirmed by the presence of significant differences between groups in gait timing (double and single support time, step and swing time) and gait regularity (both step and stride) on all three axes (Supplementary Table 1). This could overcome the need to train and deploy individual models for specific pathologies. In this study, we included several independent variables to predict the mDGI score, including information about participant demographics, disease duration, and type of assistive device, which are known to be associated with dynamic balance^17, 22^ and fall-risk assessment^23, 24^. In addition, we included a set of clinical and wearable sensor-based features descriptive of gait spatio-temporal aspects (i.e. 6MWT score, duration of stride, step, single support and double support phases) and gait quality (i.e., movement intensity and smoothness, stride/step regularity and variability, gait symmetry, and local dynamic instability)^25–27^, which are known to be impaired in PwND^3, 19, 28–32^. Alterations in gait spatio-temporal parameters have been well-documented in MS^33^, PD^34^ and ST^35^. In contrast, the quantification of factors related to gait quality has only recently attracted interest; compared to the spatio-temporal factors, the aspects are (1) more robust to differences in test settings^27^, (2) more sensitive to mild impairments^36^, (3) more responsive to rehabilitation effects^37^, and (4) more strongly associated with patient-reported walking ability^38^.

Linear models can associate *importance levels* with the features, retaining transparency in model building (e.g., $\beta$ coefficient in regressions and test statistics in group comparisons). However, such techniques do not yet provide a patient-wise estimate of the features’ contribution to the predictions. For this reason, embedding the elastic net regularised regression with SHAP results in translatable models, fostering the trust of clinical operators and the interpretability of mistakes. Regarding the most important features according to SHAP (Fig. 3), the 6MWT score had the highest impact on the prediction of the mDGI score and confirmed the strong positive correlation between the 6MWT and clinical balance measures (i.e., DGI, Berg Balance Scale, MiniBESTest, and TUG test) previously found in persons with MS^39^, PD^40^ and ST^41^. Although the 6MWT assesses walking endurance, its score can be considered as an estimate of sustainable gait speed over long periods of time. Thus, these result enforce the importance of gait speed as “sixth vital sign”^42^ that is associated with several health-related considerations, including dynamic balance and falls^43^. The use of an assistive device was the second most important feature in mDGI score prediction. This result was expected, since the level of assistance represents one of the mDGI clinical sub-scores. However, it should be highlighted that a previous study on people with neurological disorders^17^, showed that the IMU-based measures describing locomotion during the mDGI tasks (1) indicated greater impairment in persons using assistive devices than in those who do not, and (2) significantly correlated with mDGI and TUG scores. Combined, these results strengthened the association between walking aids and impaired gait patterns and, as a consequence of this correlation, poorer dynamic balance. Furthermore, since assistive devices are often adopted whenever a dynamic balance disorder occurs, it is reasonable to speculate that assistive devices are associated with a higher fall risk.

Six of the 10 most important contributors were descriptive of gait quality (i.e., regularity, intensity, and dynamic instability), further highlighting the importance of these variables in the characterization of locomotion and dynamic balance. The role of these parameters in the mDGI prediction persisted despite the strong contribution of walking speed (6MWT), suggesting that, despite their dependency on gait velocity^44^, these metrics provide different additional information. The latter demonstrates that in order to retain sufficient dynamic balance, a fine control of body links’ position or velocity is a crucial necessity, complementary to having a consistent walking speed. Similarly, Carpinella et al. found statistically significant correlations between gait quality metrics and the mDGI—Item 8 subscore, even after correcting for gait speed^45^.

Among the gait quality metrics, high stride regularity computed from VT and AP trunk accelerations provided positive contributions to the mDGI prediction. This result is consistent with findings in frail older adults, whose balance and locomotor impairments were characterized by higher inter-stride trunk acceleration variability (i.e., lower stride regularity) in the AP and VT directions^46^. The authors speculated that this finding could be related to the inability of frail subjects to generate a consistent and well-balanced propulsion of the body in the sagittal plane. This hypothesis could also apply for PwND. Significant correlations between stride regularity metrics and balance measures have already been found in previous studies of the elderly^47^ and PwND^45, 48, 49^, confirming that gait regularity is a potential rehabilitation target for improving dynamic balance and gait adaptability. Movement intensity in the AP direction (Acc. RMS$_{AP}$) was also a strong positive contributor to the mDGI predicted value—i.e., the higher the trunk AP acceleration—the better the dynamic balance and the gait adaptability. This result is supported by previous studies that find a reduction of movement intensity in persons with PD, ST, and MS^50–52^ (characterized by balance deficits) compared to healthy controls. Considering the strong correlation between trunk acceleration and walking speed^44^, a preliminary explanation for the above result could be that the trunk acceleration is positively associated with dynamic balance because gait velocity is positively associated with dynamic balance. However, a reduction in movement intensity has been previously found in people with MS compared to healthy subjects walking at a comparable speed^53^. Moreover, in the present model, Acc. RMS$_{AP}$ represented the fourth most important factor, despite the strong contribution of gait speed (6MWT). A second hypothesis can thus be formulated: people with neurological diseases may minimize upper body motion (i.e., trunk acceleration) during walking in an effort to compensate for lower limb impairments (by damping perturbations of the impaired limb) and maintain dynamic stability^50^. The local dynamic instability of gait also provided a strong contribution to the mDGI prediction. This result indicated that poorer dynamic balance is associated with higher local dynamic instability, i.e., the locomotor system has greater difficulty coping with small spontaneous perturbations caused by environmental factors (e.g., uneven surfaces) or internal factors (e.g., neurocontrol errors)^54^. A previous study of early-stage, nondisabled people with MS found that the AP Lyapunov exponent significantly correlated with clinical balance scales such as the Fullerton Advanced Balance Scale and the TUG^28^. The present findings reinforce this result and demonstrate that local dynamic instability of gait is associated with dynamic balance; this finding is also true for severely impaired persons with different neurological conditions and can thus be considered a valid quantitative measure of balance control during walking. Regarding temporal features of gait, only stride and single support duration have been found among the 10 most important factors for mDGI prediction. In particular, prolonged stride (i.e. reduced cadence) and shorter single support phase (i.e. longer double support duration) are associated to poorer dynamic balance, in line with the typical protective cautious strategy adopted to compensate for balance impairment and maintain a stable gait^45^. As shown in Fig. 3, gait symmetry, as measured by improved harmonic ratios (iHRs), provided only a minor contribution to the mDGI score estimation, suggesting that this aspect has a stronger association with the energy efficiency of gait than with dynamic balance^55^.

In summary, this work demonstrates that measures descriptive of the short steady-state walking bouts composing the 6MWT include information sufficient for predicting dynamic balance and gait adaptability to external demands and indicate that the two assessments are not mutually independent. Furthermore, since the mDGI measures the walk adaptability to different environments, it is reasonable that better quality of gait results the focal point of a prompt adaptation, and thus of a healthier walk. These results may inform rehabilitation by indicating the most important features to be addressed during balance training. The deployed models were trained on data recorded during the central 10 strides of a 30-m hospital hallway repeatedly travelled by the subject for 6 min under the supervision of a physiotherapist, as required by the 6MWT. Although this type of walking is not equivalent to that used in everyday life, these results provide a promising basis for future studies that test the possibility of extending the current model validity to more general free walks, including short steady-state walking bouts extracted from a typical stroll. Recently developed technologies based on portable devices already permit the acquisition and processing of data during unconstrained walking and provide results immediately following the recording^56^ or even in real-time^57, 58^. Regarding the specific topic of this study, the implementation of a dedicated smartphone app (one that embeds the proposed model with all data-processing procedures) able to automatically send the results to clinicians could allow a repeated assessment (self-administered or minimally supervised by a caregiver) of dynamic balance and gait adaptability in ecological settings during walking tasks that are easily sustained by PwND. Use of such an app would permit increased assessment frequency, allow tracking of performance evolution caused by the disease or by the effects of rehabilitation/pharmacological treatment, and complement in-clinic examinations. One of the study’s limitations is the retrospective and monocentric nature of the available data. Thus, given the retrospective nature of the data, the predictive power of IMU-related features on individual subitems of the mDGI scale could not be assessed. The latter would provide insights on the relationship between specific gait determinants/impairments and the different domains of the dynamic equilibrium concept. Moreover, in the proposed model, the strongest contributor to the prediction is gait speed, here estimated with the 6MWT score, which is commonly recorded by the physiotherapist in clinical practice but which is not available in real-life contexts. Future developments of the present approach should include the estimation of gait velocity from inertial sensors on the trunk^59^ or on the lower limbs^60, 61^. Another limitation is that the instrumented measures computed from the IMU were related to the central 10 strides of straight-line corridors only, thus excluding indexes descriptive of turns. This choice was made based on previous studies^26, 50, 62^ and on published results on healthy young adults^63^, which demonstrated that some of the considered gait quality measures (e.g., harmonic ratio) are affected by directional changes; to the best of our knowledge, however, the effect of turns on other indexes, such as temporal, smoothness, and intensity parameters has not been previously studied so far. Moreover, no data are available regarding the effect of turning while walking on gait parameters in people with neurological disorders. Nevertheless, considering the high impact of turning on balance and falls^64, 65^, the inclusion of measures descriptive of turns (e.g., turning speed and duration) could further improve the mDGI prediction. Future studies should address this issue and analyse the reliability of the present IMU-based assessment using a different group of 10 strides from the patient’s 6MWT. Furthermore, even if the classifiers work well on average, the predictions include outliers. Nevertheless, from a methodological point of view, the SHAP analysis confirms that these outliers did not result from any consistent bias in the prediction and thus were not caused by a systematic error observable in our feature set. The latter, together with the nested cross-validation approach that simulates the testing of new patients entering the model, confirms the reliability of the results. From a clinical point of view, the presence of outliers with an mDGI estimation error greater than 5 points could lead to misinterpretation of the results. For example, if the difference between the mDGI scores collected at two distinct times is greater than 5 points, an actual significant change in performance or a prediction error could be the cause. To mitigate this issue, it is essential that the portable device embedding the prediction model automatically and immediately send the results to the clinician so that the clinician can analyse them and, if necessary, organise an in-clinic examination. However, further analysis concerning the use of a single IMU sensor and its optimal placement is needed before the algorithms are embedded within a smartphone app. In conclusion, we deployed a model using ML techniques to predict the mDGI score in a cohort of patients with neurological impairments and obtained similar accuracies across pathology groups. The nested cross-validation approach ensured that, within the outer leave-one-subject-out test loop, every patient was assigned to the test set once. Thus, the inner k-fold cross-validation loop, used for hyperparameter optimization, avoids train-dev-test contamination for the choice of model parameters. Furthermore, our approach targeted an estimation of the continuous mDGI value instead of classifying groups according to their risk of fall^66, 67^. This approach allows researcher and clinicians to post-process regression predictions and derive a classification by imposing error boundaries; the inverse is not possible. This valuable tool helps bridge the gap between traditional balance and gait-supervised assessments in clinical settings and automated, self-administered evaluations in real-life contexts, with the aim of reducing the time and costs necessary to track disease evolution or treatment effects.

## Methods

### Participants

Ninety-five people suffering from neurological diseases were recruited from the IRCCS Fondazione Don Carlo Gnocchi (Milan, Italy). The cohort was composed of 51 people with multiple sclerosis (MS), 25 people with Parkinson’s disease (PD), and 19 people post-stroke (ST). To be included in the study, participants had to be between 20 and 85 years old; be able to walk for 20 m, even with an assistive device; and have a mini-mental state examination (MMSE) score of $\ge$ 21. People with MS were included only if they had a certain diagnosis and had not relapsed in the previous two months. People with PD were enrolled only if their Hoehn and Yahr score was < 4. Post-stroke participants were included only if the time post-onset was > 2 months. Exclusion criteria included an inability to understand and sign the informed consent, the presence of a psychiatric complication, or major cardiovascular or visual disorders. All participants signed a written informed consent to participate in this study (conformed to the Declaration of Helsinki). All methods were approved by the Ethical Committee of IRCCS Fondazione Don Carlo Gnocchi, Milan (ref 29-03-2017 and 13-02-2019). All procedures were performed in accordance with the relevant guidelines and regulations.

### Data collection and feature extraction

**Figure 4:**
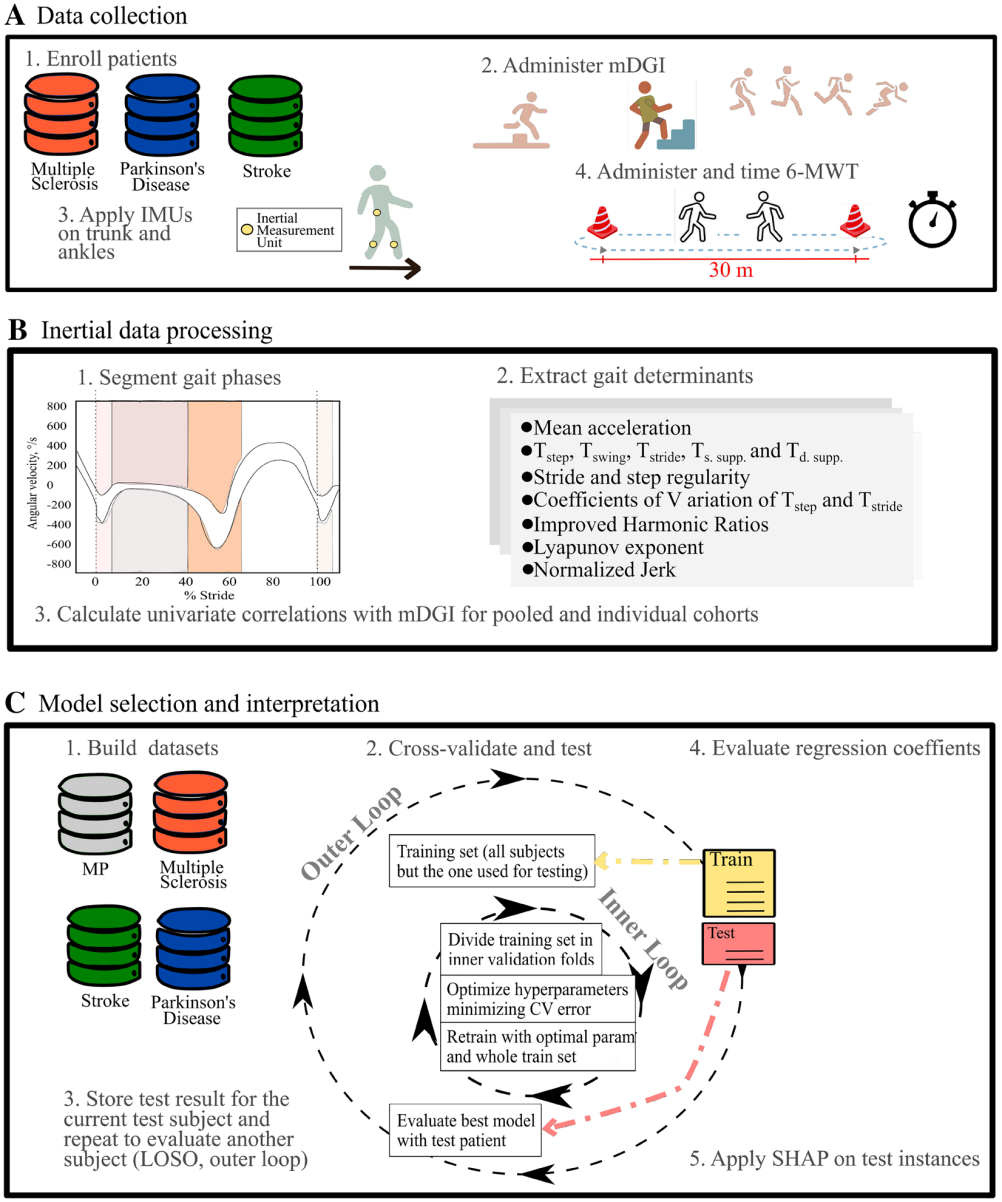
Model pipeline. In panel (**A**), steps of the data collections protocol are reported. In panel (**B** and **C**), respectively the pre-processing steps and model deployment loops are presented.

The mDGI was administered by experienced physical therapists (Fig. 4, panel A)^14^. It consists of 8 items (e.g., walking with head turns, walking around or over obstacles, stairway walking). Each item is evaluated on the basis of three aspects: gait pattern (subscore: 0–3), level of assistance (sub-score: 0–2), and time (subscore: 0–3). The mDGI total score (i.e. the sum of the sub-scores of all item), ranges from 0 to 64, with increasing values indicating better performances. In PwND, the amount of previous activity could have an impact on the following performances; thus, the participants were required to rest after mDGI execution. The resting time was determined by the patient and supervised by the physiotherapist. The participants performed the 6-minute walk test (6MWT^68^), that measures walking endurance. The test required walking back and forth along a 30-m corridor for 6 min at a fast but safe speed. If needed, the participant could use an assistive device. The presence of an assistive device was here coded as monolateral, bilateral, or no support. Participants with PD were tested while they were on-phase during antiparkinsonian therapy, approximately two hours after medication intake. The distance covered over 6 min was recorded by the examiner and represented the clinical score of the test. The participants executed the 6MWT wearing three IMUs (MTw, XSens, NL) secured to the lower trunk (L5 level) and shanks, about 20 mm above the lateral malleoli. The trunk sensor was placed on the lower back since this position is the most commonly used, according to the literature^69^. The position of the shank sensors was chosen as it was the one associated with less sensor instability, due to soft tissue artifacts. In particular, report of a decrease of 4–51% artifacts were found compared to placing IMUs in other parts of the shank or of the feet^70^. The sensors were fixed to the body by elastic bands with Velcro strips, which can also be easily applied by the subject autonomously or with the help of a caregiver. Three-dimensional accelerations and angular velocities were recorded from the three IMUs at a sampling frequency of 75 Hz; this frequency was considered adequate for the purposes of the present study since it was within the range of sampling rates (25–1000 Hz) used in previous studies^69, 71^. Then trunk accelerations were reoriented to a horizontal–vertical coordinate system^72^. Only the short steady-state walking bouts, represented by 10 consecutive strides in the middle of each corridor, were considered for the subsequent analysis, after discarding the portions of signals pertaining to the 180$^\circ$ turns at the end of each 30-m hallway^73^. Foot-strike and foot-off events were computed from the angular velocity around the medio-lateral axis of each shank^74^. Next, temporal gait determinants such as mean stride, step, and swing time, and single and double support duration were calculated. These metrics were chosen because they represent traditional measures of gait and show well-documented impairments in PwND^33–35^. Furthermore, for the step and stride times, the respective coefficients of variation were added to the dataset, as measures of step and stride variability, which are usually higher in PwND compared to healthy subjects^75^. Then, a set of 18 metrics was computed, from all trunk acceleration components (antero-posterior, medio-lateral and vertical), to provide information about gait quality domains (i.e., intensity, regularity, symmetry, stability, and smoothness) proposed by previous literature^27–32^. Gait intensity was quantified through the root mean squared value of the acceleration^27^. The gait regularity domain was represented by stride and step regularity indexes computed, respectively, as the second and the first peaks of the unbiased autocorrelation function calculated from each acceleration component^76^. Gait symmetry was quantified by the improved Harmonic Ratio (iHR) computed following Pasciuto et al.^62^. The stability domain was represented by the short-term Lyapunov exponent. This metric quantifies the local dynamic (in)stability of gait, which reflects the capability of the locomotor system to cope with small perturbations naturally present during walking, such as external disturbances or internal control errors^54^. The short-term Lyapunov exponent was calculated over the duration of one step, as fully detailed elsewhere^28^. Briefly, trunk accelerations related to ten consecutive strides in the central part of each walking bout were re-sampled to 1000 frames (10 strides $\times$ 100 frames)^54, 77^ to maintain equal data length across walking bouts and participants. This procedure was applied for the computation of the short-term Lyapunov exponent only, since this parameter is highly influenced by signal length^54, 77^. Hence, the short-term Lyapunov exponent was computed following the Rosenstein method^78^, with m = 5 and T = 10 samples (m and T were estimated using published algorithms^79^). Increasing values of the Lyapunov exponent reflect the decreasing ability of the locomotor system to manage small perturbations, thus indicating greater dynamic instability. Finally, the gait smoothness domain was quantified via the logarithm of jerk (first time-derivative of the acceleration), normalized with respect to stride duration and mean acceleration^80^. All parameters were computed for each short steady-state walking bout (10 strides) derived from the 6MWT; the median values over the whole test were then calculated to reduce the effect of possible outliers. In the present cohort, the number of walking bouts was always greater than or equal to 3. The above gait quality metrics were chosen because they showed a statistically significant correlation with the mDGI score (see Table 2) and because previous literature has demonstrated their robustness to different test settings^27^, their sensitivity to subtle impairment^28, 36, 38^ and to rehabilitation effects^37^, and their ability to discriminate between different levels of disease severity^50, 81^.

### Statistical analysis

No missing values were present in the dataset, and no data imputation techniques were adopted. To initially evaluate univariate correlations between IMU-based features and the mDGI, all extracted features were subject to Spearman’s correlation analysis, with the dependent variable set to the mDGI. To evaluate whether categorical variables had an effect on the mDGI, binary (e.g., sex) and multi-class (e.g., presence of an assistive device) categorical variables were subject to a Mann–Whitney test and a Kruskal–Wallis test, respectively. This procedure was performed for the pooled cohort and the three single-pathology cohorts. Lastly, to assess whether different pathology groups were associated with different biomechanical determinants, a group analysis (Kruskal–Wallis test) was performed, with independent variables set to the IMU-derived features and grouping variables set to the pathology (MS, PD, ST). The significance threshold was set to 0.05.

### Model selection and cross-validation

To avoid train-test contamination, all extracted features entered subsequent ML models. Namely, a regularised type of regression, the elastic net (EN), was implemented. The EN combines the penalties of the LASSO and Ridge regressions^82^, overcoming their respective implementation problems. Ridge adds quadratic regularisation via L2 penalties, assigning a non-zero coefficient to all features in the model and keeping the coefficients even if the corresponding independent variable is irrelevant to the prediction. Conversely, LASSO regression is known to deteriorate with multicollinear independent variables^83^ but neglects specific features. Elastic net combines feature elimination from LASSO and coefficient reduction from Ridge and improves on both, yielding regression parameter estimates as follows:

1 $$\begin{aligned} \hat{\beta } = argmin_{\beta }\{||y-X\beta ||^{2} + \lambda _{2} ||\beta ||^{2} +\lambda _{1}||\beta ||_{1}\} \end{aligned}$$

The special cases $\lambda _{2} = 0 \; \lambda _{1} \ne 0$ and $\lambda _{1} = 0 \; \lambda _{2} \ne 0$ correspond to the LASSO and Ridge regressions, respectively, therefore including both LASSO and Ridge in the EN model hypothesis space. In the Scikit-Learn implementation

2 $$\begin{aligned} \hat{\beta } = argmin_{\beta }\left\{ \frac{1}{2N_{samples}}||y-X\beta ||^{2} + \alpha \, l1_{ratio}||\beta ||_{1} +\frac{\alpha }{2}\left( 1-l1_{ratio}\right) ||\beta ||^{2}\right\} \end{aligned}$$

the $l1_{ratio}$ describes the tendency toward a LASSO regularization ($l1_{ratio} \sim 1$) or a Ridge regularization ($l1_{ratio} \sim 0$) and the $\alpha$ acts a scaling parameter of the regularization process.

To maximize robustness of the model, we implemented a nested cross-validation approach. In brief, such an approach consists of two k-fold cross-validation loops: an outer loop identifies the test set for each of its folds, while the inner loop implements a further split of the dataset for training and validation^84^. In the outer layer (testing layer), a LOSO testing procedure was used (Fig. 4, panel C). Specifically, one patient at a time was withheld for testing and the remaining N − 1 were used for training and cross-validation. The N − 1 patient were resampled using the Synthetic Minority Oversampling Technique for Regression (SMOTER^85^), generating training and validation samples equally distributed across the mDGI range. Then, the resampled training set was used to cross-validate and optimize the model’s hyper-parameters by minimizing the cross-validation median absolute error $E_{val} = \frac{\sum _{k=1}^{3} Accuracy_{k}}{K_{inner\, folds}}$ and averaging the accuracies across the K inner folds (Eq. 3). For the EN regression, $\alpha$ and $l1_{ratio}$ were optimized together with the majority class down sampling rate ($\%d$) and the minority class synthetic samples generation rate ($\%o$)^85^. Specifically, both percentages were allowed to vary between the 200 and 800%. The number of inner folds (*K*) used to optimize all of the aforementioned parameters was set to 3. Then, with optimal hyper-parameters, EN regressions were retrained on all the $N-1$ training patients, including the synthetic samples. Lastly, the model was tested on the patient withheld from the outer split. This procedure was repeated for the multi-pathological cohort (MP) and the single pathology cohorts ($SP_{MS}$, $SP_{PD}$ and $SP_{ST}$) and the predictions on the outer test samples were stored and aggregated (Fig. 4). The described pipeline was repeated using three different datasets: (1) IMU-derived features and clinical variables (6MWT score, presence of assistive devices), (2) clinical variables only (Supplementary Fig. 1), and (3) IMU-derived data only (Supplementary Fig. 2). All machine learning pipelines were implemented with the Optuna and Scikit-Learn libraries.

### Explainable elastic-net

Generalised linear models already enable interpretability and explainability measures by assigning to each independent variable k the magnitude of its related regression coefficient k and therefore calculating the effect of all features on the prediction via the dot product $\cdot$x. Nevertheless, given the nested cross-validation implementation, in the outer split, each patient is included N 1 times in a training set and only once in a test set. Consequently, N models trained on the N permutation of N 1 patients will result in N parameter estimates (K, N). Accordingly, evaluating feature importance by averaging the N-coefficients is possible but has two major drawbacks. First, the resulting variability in the parameter estimates can be relevant. Second, estimates are derived from mean trends in the training subset and are not patient-specific. SHAP overcomes these limitations by determining feature contributions to the prediction specifically for the individual subjects, resulting in one value per subject per feature^20, 21^.

### Model comparison

Wilcoxon signed-rank tests were applied between the MP model and the aggregated predictions of the SP models. Furthermore, individual SP models were also compared to the predictions of the same instances made by the MP model with Wilcoxon signed-rank tests.

## Supplementary Information

Supplementary Information 1.

Supplementary Information 2.

## Data Availability

Data has been provided with the manuscript and codes can be made available upon request to Piergiuseppe Liuzzi and Ilaria Carpinella for replication purposes only.

## Acronyms

mDGI: Modified dynamic gait index
PwND: Persons with neurological diseases
TUG: Timed up and go
MS: Multiple sclerosis
PD: Parkinson’s disease
ST: STroke
6MWT: 6-Minutes walk test
IMU: Inertial measurement units
MP: Multi-pathology
AP: Antero-posterior
VT: VerTical
ML: Medio-lateral
SHAP: SHapley additive explanations
MDC: Minimally detectable change
