# Supplementary material for: Machine learning based estimation of dynamic balance and gait adaptability in persons with neurological diseases using inertial sensors
Source: Sci Rep. 2023 May 27;13:8640. doi: 10.1038/s41598-023-35744-x (PMC10224964; doi:10.1038/s41598-023-35744-x)
Supplement: Supplementary file 1 — Supplementary Information 1. [file 41598_2023_35744_MOESM1_ESM.pdf]

Supplementary Materials

Supplementary Figure 1

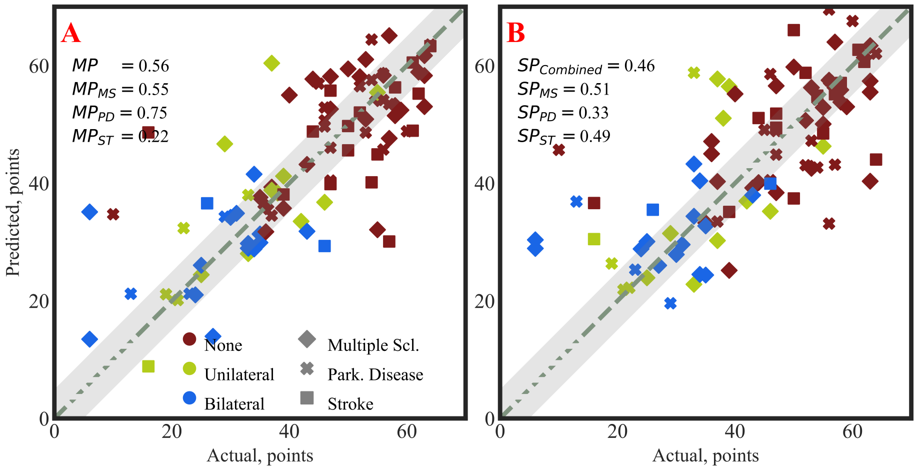

Figure 1: Model predictions with only 6MWT score and presence of an assistive device as training data. The pipeline used is the same to obtain Figure 2.

Supplementary Figure 2

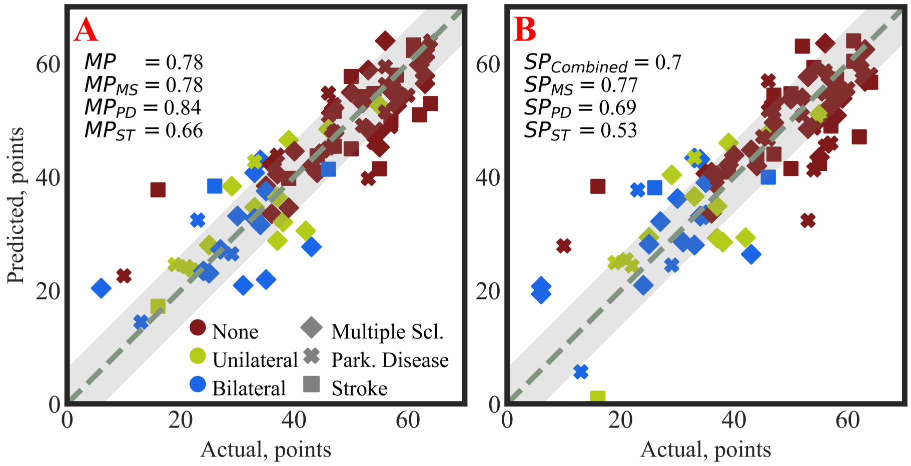

Figure 2: Model predictions with only IMU-related data. The pipeline used is the same to obtain Figure 2.

Supplementary Table 1

Group comparison between IMU-derived features across different pathological groups (MS, ST, and PD). The test adopted is the Kruskall-Wallis test with grouping variable set to the pathological group and independent variable set to the IMU-derived feature.

Variable Name	χ	p-value
6MWT	1.959	0.376
T_{stride}	4.367	0.113
**T_{swing}**	**6.657**	**0.036**
**$T_{d,support}$**	**8.135**	**0.017**
**$T_{s,support}$**	**10.556**	**0.005**
**T_{step}**	**10.694**	**0.005**
**Step Regularity$_{ap}$**	**8.486**	**0.014**
**Stride Regularity$_{ap}$**	**6.611**	**0.037**
Step Regularity$_{ml}$	3.649	0.161
**Stride Regularity$_{ml}$**	**10.043**	**0.007**
**Step Regularity$_{vt}$**	**6.070**	**0.048**
**Stride Regularity$_{vt}$**	**9.773**	**0.008**
$CV_{T,stride}$	3.111	0.211
$CV_{T,step}$	3.056	0.217
iHR$_{ap}$	5.476	0.065
iHR$_{ml}$	5.650	0.059
iHR$_{vt}$	3.975	0.137
**Lyapunov step$_{ap}$**	**11.398**	**0.003**
Lyapunov step$_{ml}$	1.550	0.461
Lyapunov step$_{vt}$	0.225	0.893
RMS Acc.$_{ap}$	0.626	0.731
RMS Acc.$_{ml}$	5.505	0.064
RMS Acc.$_{vt}$	1.703	0.427
Log. Norm. Jerk$_{ap}$	0.122	0.941
Log. Norm. Jerk$_{ml}$	1.059	0.589
Log. Norm. Jerk$_{vt}$	5.394	0.067

Legend. 6MWT: 6-Minutes Walk Test; T: Time; CV: Coefficient of Variation; RMS: Root Mean Square; AP: Antero-Posterior; ML: Medio-Lateral; VT: Vertical.
